# Artificial intelligence for cardiac imaging is ready for widespread clinical use: Pro Con debate AI for cardiac imaging

**DOI:** 10.1093/bjro/tzaf015

**Published:** 2025-06-06

**Authors:** Domenico Mastrodicasa, Marly van Assen

**Affiliations:** Department of Radiology, University of Washington School of Medicine, Seattle, WA, 98105, United States; Translational Lab for Cardiothoracic Imaging and Artificial Intelligence, Department of Radiology and Imaging Sciences, Emory University, Atlanta, GA, 30322, United States

**Keywords:** cardiac imaging, artificial intelligence, clinical implementation

## Abstract

Artificial intelligence (AI) has made significant strides in cardiac imaging, offering advancements in image acquisition, risk prediction, and workflow automation. However, its readiness for widespread clinical adoption remains debated. This review explores both sides of the argument across key domains. It discusses the advantages and challenges of AI for cardiac imaging regarding pre-and post-processing, risk-stratification and prognostication, workflow augmentation, regulatory and ethical frameworks, and cost-effectiveness of AI tools. It will discuss the diagnostic accuracy shown by AI for automated measurements, improved image quality and workflow efficiency with AI-driven worklist prioritization. The potential of personalized care using AI-based prognostic models. It discusses regulatory frameworks for approving AI tools, while ethical frameworks to ensure safe and ethical use of AI are being implemented, simultaneously reimbursement is becoming available, signalling growing trust in their safety and efficacy. It also addresses the challenges AI has yet to overcome, such as the lack of generalizability across diverse populations, limited availability of outcome data and cost-efficacy studies. Despite progress, regulatory and ethical frameworks still struggle to keep pace with AI’s rapid evolution, raising concerns about accountability, patient safety, bias, data privacy, and algorithmic transparency.

## Introduction

**Figure 1.**
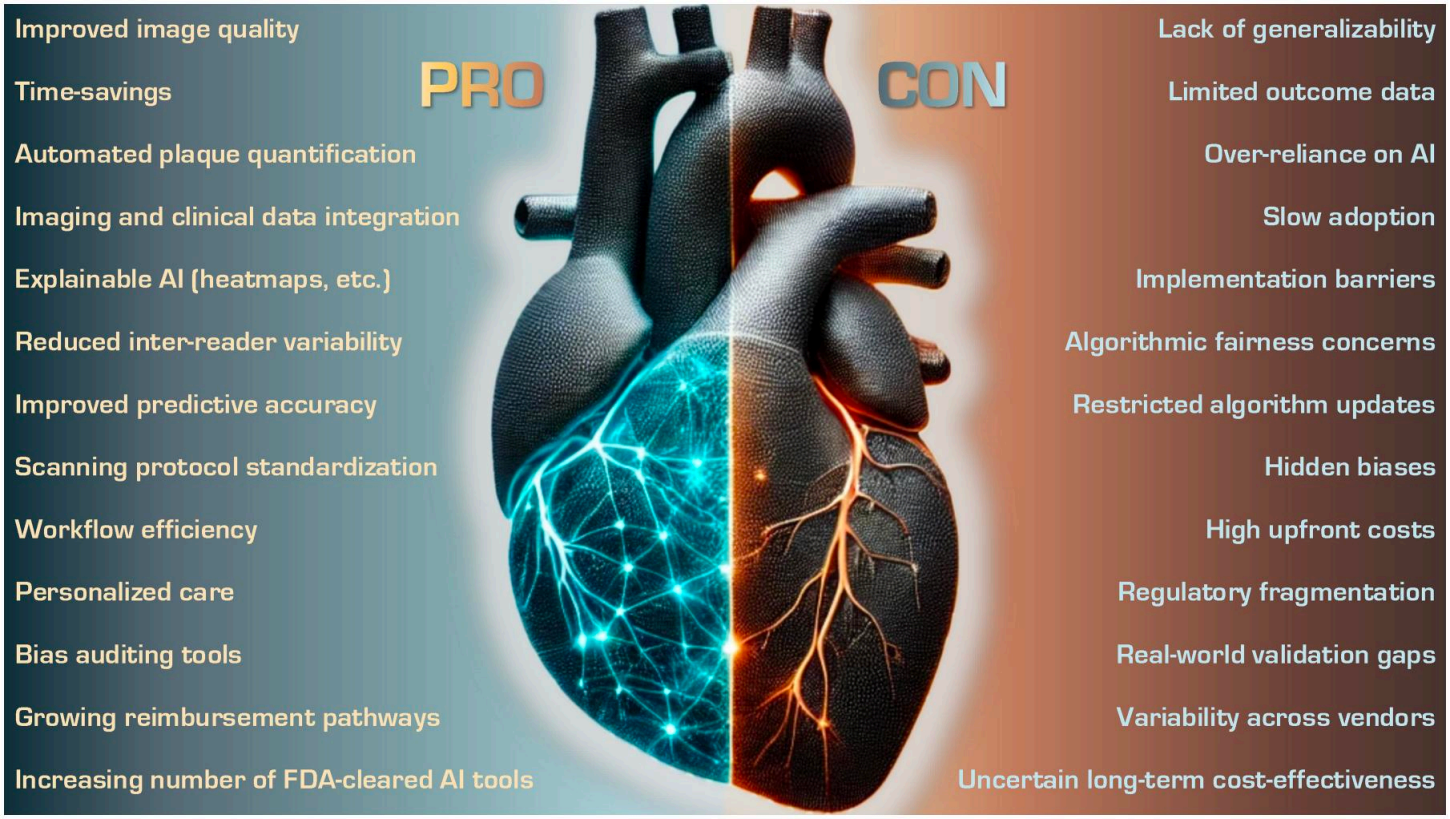
Overview of Pro and Con arguments for the clinical use of AI in cardiac imaging.

Artificial intelligence (AI) has made significant strides in cardiac imaging, particularly in CT and MRI, offering advancements in image acquisition, risk prediction, and workflow automation. Radiology is the largest contributor to FDA-approved AI applications (76.9%), and circulatory system applications represent 20.8% of approvals.^1–3^ The field of cardiac imaging has grown increasingly complex due to the need for high temporal and spatial resolution to visualize atherosclerotic plaques on coronary vessels and to distinguish subtle tissue structural differences in the myocardium. Addressing this complexity is critical as cardiovascular disease remains the number one contributor to global mortality.^4^ AI offers a powerful means to navigate these challenges, with applications available throughout the cardiac imaging workflow, including appropriate imaging selection, diagnosis, prognosis, and risk assessment.^5^^,^^6^ Determining whether AI is ready for widespread clinical use in this domain is essential, as it involves assessing its reliability, generalizability, and clinical impact in real-world settings. The question of whether AI can fully meet the stringent requirements for clinical deployment—particularly in terms of safety, accuracy, and equity across diverse populations—remains a critical debate within the medical and technological communities. AI can improve diagnostic accuracy and patient outcomes, but premature deployment could undermine trust and lead to unintended clinical consequences. This review aims to critically discuss the readiness of AI for clinical deployment in cardiac CT and MRI in the form of a debate, weighing the potential benefits and challenges to help determine if the time has come for AI to be a routine part of clinical practice, with broad implications for all stakeholders. For clarification purposes, Table 1 gives an overview of main AI terminology and their definitions, while Figure 1 gives and overview of pro and con arguments discussed in this review.

**Table 1.** AI definitions and terminology.

Term	Definition	Use case
**Artificial intelligence** **(AI)**	Refers to the simulation of human intelligence by machines, typically through processes such as learning, reasoning, and problem-solving.	
**Machine learning (ML)**	A subset of AI that involves algorithms learning from data to make predictions or decisions without being explicitly programmed for the task.	
**Deep learning** **(DL)**	Subset of ML that uses neural networks with multiple layers (deep networks) to learn from large amounts of data.	
**AI algorithm**	An AI algorithm is computational procedure used to solve a problem or perform a task in AI.	
**AI model**	Mathematical framework resulting from training an algorithm on data, allowing it to make predictions or decisions. Unlike an AI algorithm alone, an AI model is shaped by the patterns it has learned from the data it was trained on.	
**Reliability**	The ability of an AI system to consistently produce the same results under consistent conditions.	Reliable AI models are crucial in fields like healthcare, where consistent results are essential for diagnosis.
**Reproducibility**	The degree to which an AI model’s results can be replicated by different researchers or under different conditions using the same data and methods.	In scientific research, reproducibility ensures that AI models can be trusted for validation and further study.
**Bias**	Systematic error introduced into AI models due to skewed or unrepresentative data, leading to unfair outcomes or performance disparities.	Bias in AI can result in discriminatory outcomes healthcare diagnosis and treatment recommendations.
**Accuracy**	The measure of an AI model’s performance, indicating how well the model’s predictions or classifications match the true values.	Accuracy is a key metric for AI models in tasks like disease diagnosis or object recognition.
**Black box**	A term used to describe AI or ML models, especially deep learning models, where the internal workings are not easily interpretable or understandable by humans.	Black-box AI models are often used in healthcare, but their lack of transparency can be a drawback.
**Training**	The process of teaching an AI model by feeding it data and allowing it to learn the patterns and relationships within the data.	Training a model to recognize different types of cardiac anomalies from imaging datasets.
**Testing**	Evaluation of the AI model’s performance using new, previously unseen data to determine its accuracy and reliability.	Testing an AI model for cardiac CT and MRI scans from a separate dataset from the training data (i.e. different scanners)
**Validation**	Tuning model parameters and selecting the best model, often involving the use of a validation dataset. Not to be confused with an external validation set.	To optimize the AI model performance before deployment in the testing set.
**Hyperparameters**	The settings or configurations that are used to control the learning process of an AI model.	Adjusting hyperparameters like learning rate to improve the performance of an AI model in cardiac imaging.
**External validation**	The evaluation of an AI model using completely independent data not used during the training or testing phase. Not to be confused with the validation set during AI development.	External validation is used to assess whether an AI model generalizes well across different populations and settings.
**Cross validation**	A technique used to assess the generalizability of an AI model by splitting the data into multiple subsets and training/testing the model across different combinations of these subsets.	Cross-validation ensures that the model is robust and reduces overfitting.

## Is AI ready for pre- and post-processing of cardiac imaging?

### Pro

Coronary computed tomography angiography (CCTA) allows non-invasive detection of subclinical coronary artery disease (CAD).^7^ Several clinical trials have indicated CCTA as a gatekeeper for invasive coronary angiography.^8–12^ CCTA can identify individuals who may benefit from coronary revascularization while accurately ruling out significant CAD in low-to-intermediate risk populations. This clinical workflow reduces unnecessary invasive studies and leads to cost savings and fewer complications.^13^ However, accurate evaluation of luminal stenosis and characterization of coronary plaque composition rely on high image quality and remains a time-intensive task prone to inter-reader variability, potentially leading to inconsistent clinical decisions and impacting patient outcomes.^14^^,^^15^ Studies have reported improved image quality using AI for image reconstruction,^16^^,^^17^ leading to increased vessel sharpness, improved accuracy of stenosis grading, and better stent visualization.^18^^,^^19^ AI tools can automatically identify and quantify luminal stenosis^20^^,^^21^ and categorize patients according to a standardized reporting framework that guides clinical management, CAD-RADS.^15^^,^^22^^,^^23^ This level of automation can improve accuracy and consistency for CAD assessment. Studies have reported strong agreement between AI tools and manual scoring for quantifying plaques with different phenotypes (calcified vs non-calcified).^10^^,^^14^^,^^15^^,^^21^ AI-based approaches reduce analysis time and inter-reader variability by automating coronary segmentation and plaque quantification.^23^^,^^24^ AI tools also support serial assessments to track changes in plaque burden and morphology over time, providing clinically valuable insights into lipid-lowering therapy effectiveness.^25^ Additionally, AI combined with computational fluid dynamics provides non-invasive functional information through CT-derived fractional flow reserve (CT-FFR), offering insights into the hemodynamic significance of coronary lesions.^26–28^ CT-FFR has shown value in clinical practice by reducing invasive follow-up procedures.^13^^,^^29^ Clinical uptake of these AI biomarkers is reflected in the analysis of reimbursement rates, showing that CT-FFR is the most reimbursed FDA-approved AI application with over 63 000 claims between 2018-2023.^30^

In cardiac MRI, AI tools can accurately extract atrial and ventricular volumes, overall heart function, and left ventricle myocardial mass, which are key metrics needed to evaluate cardiac performance and monitor disease progression.^31–33^ These tools have been validated against manual analysis, with significant time savings.^32^^,^^34^ Commercially available AI solutions for biventricular function assessment are widely adopted in clinical practice. Tools for advanced applications such as late gadolinium enhancement (LGE) scar quantification, myocardial strain quantification, and parametric mapping are also emerging, showing good agreement with manual methods.^35^^,^^36^ Quantifying LGE scar burden is clinically relevant, as scar burden is linked to arrhythmia and mortality risk, providing important prognostic information that can influence treatment strategies, such as implantable cardioverter defibrillator implantation.^37^^,^^38^ Studies have reported that AI tools produced results comparable to expert human interpretation.^39^^,^^40^ Furthermore, AI-based methods like cardiac MRI fingerprinting enable multi-parametric mapping within a single scan, reducing acquisition times and increasing efficiency by simultaneously capturing parameters such as T1, T2, and proton density.^41–43^

### Con

The clinical reliability of coronary plaque imaging with CCTA has several limitations, including image quality and the choice of CT reconstruction algorithm, which can negatively impact the performance and reliability of AI-based analyses. Spatial resolution is another limitation for accurate plaque characterization with CCTA compared to invasive methods, particularly for small or complex coronary plaques. While retrospective studies show promise, prospective data remain scarce, leaving doubts about the ability of AI tools to improve patient outcomes and cost-effectiveness in real-world settings. A few prospective studies have emerged, particularly for CT-FFR.^13^^,^^29^ However, most AI-related cardiac imaging studies, including those based on large registries such as APOLLO and the UK Biobank, use retrospectively acquired imaging data with prospective follow-up.^44^^,^^45^ This distinction limits the strength of conclusions regarding clinical outcomes. Finally, differences in plaque quantification may result from different commercial software, lead to variability in clinical interpretation, and limit the comparability of plaque volumes.

For cardiac MRI, while AI performs well for left ventricular parameters, accuracy for the right ventricle is lower. AI-based strain assessment shows limited agreement with manual segmentation in populations with left ventricular hypertrophy. Clinical adoption of AI-driven strain analysis remains limited due to a lack of standardized approaches and variations in strain values across commercial software. Myocardial tissue characterization requires separate acquisitions, making it time-consuming and susceptible to MRI scanners and pulse sequence variations. AI-driven techniques, including cardiac MRI fingerprinting, show promise; however, their clinical application remains limited due to cardiac and respiratory motion sensitivity. AI tools for these applications have been increasingly tested and validated; however, further testing and refinement are necessary before widespread clinical use. Furthermore, integrating AI tools with existing archiving clinical systems remains technically challenging, often requiring vendor-specific solutions. Establishing robust quality control procedures and escalation protocols for AI failures will also be essential to ensure patient safety and support clinical trust in AI-assisted workflows.

## Is there sufficient clinical evidence to support AI for risk-stratification and prognostication in cardiac imaging?

### Pro

Cardiac imaging provides valuable information for risk-stratification and prognostication, which helps clinicians make informed decisions on patient management beyond clinical risk scores.^46–50^ Multiple imaging-based risk scores are routinely used in clinical practice, such as coronary artery calcium scoring (CACS) and CAD-RADS^51–55^ and AI-based coronary plaque burden quantification, as a more recent example. These biomarkers can be used to improve risk-stratification and prognostication individually.^56–58^ Besides imaging data, many risk/prognostic scores use data from different sources, such as the American Heart Association Pooled Cohort Equation/Predicting Risk of Cardiovascular Disease Events (AHA PCE/PREVENT) risk scores and the Framingham risk score.^59–62^ The accuracy of these single-modality approaches can be improved by combining clinical and imaging data. Several studies on multimodality AI have shown that combining clinical factors routinely collected in electronic health record systems (e.g. age, sex, cardiovascular risk factors) with cardiac imaging data (e.g. CACS) in AI models significantly improves the predictive accuracy for all-cause mortality compared to either clinical or imaging data alone.^48^^,^^49^^,^^63^ Most multi-source AI models combine manual or semi-automatic extracted imaging features with clinical data. Direct evaluation of pixel-based cardiac imaging by AI-based algorithms could improve prognostication and make clinical use more feasible by reducing manual input. Deep learning algorithms such as convolutional neural networks, can automatically extract biomarkers directly from imaging data, and allow automated quantification of for example CAC^64^, plaque burden^57^ and adipose tissue,^65^ which can be used for prognostication purposes. This automation step would make prognostic models clinically feasible by increasing time efficiency. Efforts are being made into using explainable AI approaches,^66^ such as total gain plots, Shapley additive explanation (SHAP) plots or heatmaps.^67^ These assist in visualizing which variables/features contribute to the predicted risk/outcome, creating more transparent algorithms. This is aimed to increase trust in AI to support clinical adoption.

### Con

Despite promising results, robust data supporting AI-based prognostication of cardiac conditions is lacking. Most prognostication studies using AI have been performed retrospectively based on data from a single centre, or very well-curated datasets, which may not represent real-world clinical settings. Currently, it is unclear how these models may affect patient care and outcomes. Keeping in mind feasibility, a combination of large retrospective validation studies with smaller prospective external validation studies is needed to provide evidence for claims made on risk-stratification and prognostication. Predictive AI models are especially sensitive to feedback loop bias, where AI is optimized based on a biased or flawed metric.^68^ In addition, any bias in AI models, whether used for risk-stratification or post-processing can perpetuate this bias to subsequent decision-making and patient care. An example is given by Puyol-Anton et al^45^ who shows that AI performance differed across race for cine segmentation led to increased heart failure misclassification rates among minority populations. Hence, prognostic AI models require robust performance evaluation and must consider fairness and equity as well as longitudinal performance.^69^ AI-based prognostication is further limited by the absence of comparison with physician-based reference standards and reduced explainability compared to more straightforward quantification/segmentation applications. Despite efforts made with regards to explainable AI, the relationship between input data recorded at one time-point and the output data recorded at another time-point is often complex and influenced by many factors and decisions in a dynamic process. These dynamic processes are difficult to grasp with explainability approaches such as the earlier described SHAP values and heat maps. Developing trust in AI prognostication will remain an issue without knowing which factors influence prediction accuracy. Additional limitations include the ability of AI models to deal with missing data, the variability of data/features used in these models, and limited information on reproducibility across vendors, populations and hospitals.

## Can AI augment and optimize the cardiac imaging workflow?

### Pro

The potential benefits of AI in cardiac imaging extend beyond image acquisition and interpretation to include operational tasks like imaging order evaluation, scanning protocol selection, and patient scheduling. Studies have shown that AI can improve traditional rule-based clinical decision support systems to suggest the most appropriate imaging exam by leveraging both structured (e.g. laboratory and imaging results) and unstructured data (e.g. clinical notes).^47^^,^^70–72^ Unlike rule-based systems that follow predefined “if-then” scenarios based on a limited number of symptoms and risk factors, AI-based systems can account for patient scenarios with more clinical variables.^70^ For example, AI can account for potential contraindications, such as chronic kidney disease in patients scheduled for contrast-enhanced studies, and recommend suitable alternative imaging tests.^71^ AI can also leverage radiology reports to identify patients likely to require several imaging studies, which may result in increased health care costs and exposure to ionizing radiation.^73^ Manually assigning scan protocols and selecting acquisition parameters are time-consuming tasks that require radiologists to pause image interpretation. In addition to disrupting the workflow, it can lead to errors and inconsistencies. Studies have reported that AI can streamline routine protocoling by analysing patient data and suggesting appropriate protocols with an accuracy of 80% or higher.^74–76^ By automating these tasks, AI reduces radiologist workload and allows them to focus on more complex diagnostic tasks. Additionally, AI-based protocoling can use prior studies, patient-specific data, and clinical indications to suggest consistent, optimal protocols, further reducing manual input and improving workflow efficiency.^70^^,^^74–76^ Assigning the correct scanning protocol positively impacts patient scheduling as well, as scanning protocols vary in time requirements. By considering factors like clinical complexity, patient needs, and staff availability, AI can also predict which patients are likely to miss their appointments, enabling targeted interventions such as automated reminders. These strategies help reduce no-shows, optimize resource utilization, and minimize wasted appointment slots.^74^

### Con

Non-diagnostic AI applications in cardiac imaging aim to reduce human error and increase radiologist efficiency while improving patient safety and satisfaction. However, much of the current evidence supporting these applications comes from general radiology settings, which may not fully reflect the unique challenges of cardiac imaging. A potential drawback of AI-based imaging orders is the risk of over-reliance on automated recommendations. In complex cases requiring nuanced clinical judgement, excessive dependence on AI could erode a clinician’s ability to make independent decisions, similarly to what happens for the automation bias.^77^ In high-stakes or complex clinical scenarios, such over-reliance may lead to errors of omission/commission and reduce the clinician’s engagement in independent decision-making. Studies have demonstrated that AI suggestions are often accepted without verification, even when incorrect, particularly under cognitive load or time pressure.^78–80^ These findings raise valid concerns about how AI integration may unintentionally erode clinical judgement, highlighting the need for human-in-the-loop safeguards and explainable AI strategies. In addition, while rule-based systems have limitations, they provide greater transparency compared to the “black-box” nature of AI algorithms that make the reasoning behind exam selection less interpretable, especially in challenging clinical scenarios. AI can assist in standardizing imaging protocols, but the lack of transparency in the decision-making processes behind the AI tools poses challenges in understanding the rationale behind specific recommendations. Some novel approaches, such as incorporating reasoning steps in AI outputs, currently an upcoming strategy for large language model (LLM) use, might enhance the transparency in these models.^77^

Furthermore, different vendors may implement conflicting protocols or different image sequences, which complicates achieving consistent imaging quality in health care settings with multivendor scanners. The lack of standardization among electronic systems for clinical and imaging data further exacerbates these challenges, potentially compromising AI tool performance and leading to variability in patient care. Finally, AI-driven scheduling offers efficiency but may lack the flexibility to respond to unexpected changes, such as patient emergencies. Compared to manual methods, automated systems could lead to rigid scheduling practices and an inability to adapt to real-world complexities, which will likely require human supervision. These challenges represent barriers to the widespread implementation of non-diagnostic AI tools in cardiac imaging.

## Are regulatory bodies providing clear guidelines and approvals for the use of AI in cardiac imaging?

### Pro

Regulatory bodies are increasingly recognizing the importance of AI in healthcare, providing clearer guidelines and frameworks for its approval and use. Several regulatory agencies have established pathways for AI-based medical devices, ensuring that they meet safety and efficacy standards. In the U.S., the FDA has established the Digital Health Precertification Program,^81^ which evaluates AI-based medical devices based on risk with their final report dating September 2022. The U.S. Executive Order on AI (October 2023)^82^ underscores the government’s commitment to responsible AI development, highlighting the need for accountability, standardized testing, and managing AI-related risks. Similarly, in the European Union,^83^ regulatory bodies like the European Medicines Agency are implementing rigorous guidelines through the Medical Devices Regulation and In Vitro Diagnostic Regulation, which categorize AI-based medical devices by risk. The EU AI Act (formally adopted May 2024, with full implementation by 2026) aims to create trustworthy AI frameworks, banning AI applications that exploit vulnerable populations.

Most clinical AI applications start as research-based prototypes. Correct reporting of research findings regarding these AI applications and their functionality enhances their clinical use and uptake. Therefore, a wide range of reporting guidelines exist covering the range of potential study designs and stages of AI/ML model development and testing.^20^ Many represent an evolution of existing reporting guidelines, which have been updated and refined for AI/ML applications.

### Con

The dynamic nature of AI poses challenges for traditional regulatory frameworks, which may struggle to keep pace with rapid advancements and ensure ongoing compliance. This can lead to gaps in balancing compliance with technological advancements.

For instance, the FDA only supports pre-planned intermittent AI algorithm updates and does not support continuous learning AI algorithms as of yet, as guidance on ongoing performance monitoring, evaluation, and bias assessment is lacking. EU regulations prevent modifications to AI systems altogether after approval, which may hinder the evolution of AI algorithms and limit flexibility to optimize AI algorithms for clinical use. While intended use statements are mandatory in the EU for clearance, studies have shown that 95% (*n* = 132/139) of the intended use statements analysed were incomplete, with the intended use environment and intended patient population frequently omitted.^84^

The fast-changing regulatory frameworks make long-term planning of AI development and implementation challenging due to the need to continuously adjust to changing regulations and approval processes. Additionally, international regulations vary significantly, making global deployment and multi-site studies challenging due to differing requirements and frameworks across regions hampering global commercial development and deployment.^85^

Notably, regulatory approval of AI devices does not necessarily equal good clinical performance. Most FDA-cleared AI tools were evaluated using retrospective test data, and the endpoints measured may not include side-by-side comparison of clinicians’ performance with and without the AI/ML tool. In an overview including 100 CE-marked (51 with FDA approval) AI products from 54 vendors, 64% had no peer-reviewed evidence of its efficacy.^86^ The remaining 36% of products was described by 237 papers that predominantly (65%) focused on diagnostic accuracy, 18% had evidence that validated the (potential) impact on diagnostic thinking, patient outcome, or costs. Only ∼50% of the papers (116/237) were independent and not (co-)funded or (co-)authored by the vendor. In addition, several studies have shown that performance of medical AI models underperform after deployment.^86^

## Can the potential risks and ethical concerns of using AI in cardiac imaging be effectively managed?

### Pro

Ethical concerns like transparency, fairness, non-maleficence, and privacy are increasingly being addressed by radiological and other medical societies and regulatory bodies. Guidelines and recommendations are under development for using bias auditing tools, fairness metrics, counterfactual analysis, sensitivity analysis, and adversarial testing to help identify and mitigate biases, particularly those related to race and gender. Additionally, the U.S. FDA, Health Canada, and the UK’s Medicines and Healthcare products Regulatory Agency have collaboratively developed Good Machine Learning Practices to guide the creation of AI algorithms, which helps ensure safer and fairer deployment of AI in clinical care. By using diverse datasets, AI models can be better trained to minimize bias and ensure equitable performance across various patient subgroups. Bias auditing tools can address inequities for race across risk models for a variety of diseases, including cardiac disease.^87^^,^^88^ Examples of bias auditing tools include fairness metrics, counterfactual analysis, sensitivity analysis, algorithmic transparency, and adversarial testing.^87^^,^^88^ Although disparities and bias in cardiovascular data can translate into AI performance, AI can also be used to mitigate bias. Investigations into the nature of risk-of-bias for cardiovascular disease (CVD) risk prediction demonstrated a 43% reduction in bias-effect in AI approaches compared to non-AI approaches.^89^ Strategies that led to decreased bias included using a greater number of optimization techniques, increased use of cross-validation, and more substantial performance evaluation, including operator variability analysis. AI can also be used to enrol more representative sample in clinical trials. An AI-powered tool to overcome potential bias in patient pre-screening processes for oncology trials resulted in a 24% to 50% increase in patients correctly identified as potentially eligible for clinical trial compared to standard practice.^90^

Monitoring algorithm deployment is crucial for understanding real-world performance and detecting bias early.^91–94^ A study on AI accuracy for myocardial perfusion imaging using SPECT for predicting obstructive CAD, noted that most patients serving as the reference standard were high-risk patients with invasive testing, resulting in overestimation of obstructive CAD and increased referral for invasive testing in low-risk patients.^95^ After several strategies to mitigate selection bias, they showed that augmentation of low-risk patients improved AI accuracy and had better calibration after external validation.

### Con

Despite ongoing efforts, issues such as data privacy, algorithmic bias, and lack of transparency are complex to address. Unbalanced representation in clinical data significantly impacts the performance of AI models.^96^^,^^97^ Despite evidence that cardiac biomarkers vary across populations, women and racial/ethnic minorities are consistently under-represented in cardiac clinical trials.^98^ A systematic review of 740 cardiovascular trials between 2010 and 2017 revealed that only 38% of participants were women.^99^ These discrepancies across sample representation are embedded in data used for development and validation of current AI algorithms. As a result, AI models may perpetuate biases, reducing their effectiveness for under-represented groups, and exacerbating existing disparities in health care outcomes.^100^ In addition, labelling bias also plays a role in AI development. Data has shown that implicit bias plays a role in therapy allocation in clinical practice.^101^ When this data is used as reference label in training an AI algorithm, this bias is embedded in the AI algorithm. Sex/racial bias was investigated in AI-based cardiac MRI (CMR) segmentation in the UK Biobank database,^45^ which is sex-balanced but not race-balanced. DICE similarity scores (DSC) showed significant differences between racial groups (94% for White individuals vs 86%-89% for minority ethnic groups). No covariate accounted for the observed bias in DSC among different racial groups. These errors in AI-based segmentation can affect volumetric and functional measures, potentially leading to misclassification of disease severity with major consequences for diagnosis and follow-up treatment. It is imperative to report the demographics of the data used for AI training and validation, to create awareness of these data unbalances and selective use of data for research purposes. A concern is the existence of hidden signals in medical images detected by AI, such as race and sex, which could affect performance and lead to unintended consequences.^102^

AI models trained on biased or unrepresentative data may perpetuate or even exacerbate inequalities in health care, with greater effect on under-represented groups. Incorrect interpretation of AI performance metrics can lead to the false assumption that a model works universally well across patient populations. This can result in harmful misdiagnoses or inappropriate treatments in subgroups where the model underperforms. Even though there is more attention for equitable and ethical use of AI, and guidelines and fairness tools exist,^103–105^ the rapid development of AI technologies often outpaces regulatory and ethical frameworks, leaving gaps in accountability and transparency.

## Does the cost-benefit analysis favour the widespread adoption of AI in cardiac imaging?

### Pro

The cost-benefit analysis theoretically favours the adoption of AI in cardiac imaging due to several key advantages. AI can significantly reduce time requirements for image interpretation, automate routine tasks, and enhance diagnostic accuracy, allowing radiologists to focus on more complex cases. This efficiency can lead to quicker diagnosis, potentially improving patient outcomes while reducing overall health care costs.^72^^,^^106^ Moreover, by optimizing workflows and reducing the burden on radiologists, AI can help address staff shortages and manage increasing demands for imaging services. AI-based procedures are beginning to receive medical reimbursement, especially in areas where AI has shown robust data to improve clinical outcomes, which further strengthens the financial argument for its adoption. As of 2023, with over 500 medical AI devices approved by the FDA, there were 16 billable AI procedures under 32 unique CPT codes in the United States, with usage concentrated in high-income, metropolitan areas near academic hospitals.^30^ The US Centers for Medicare & Medicaid Services (CMS)^107^ recently introduced current procedural terminology (CPT) codes for AI-based plaque quantification on coronary CTA, enabling providers to bill for this innovative technology used to assess atherosclerotic burden. This milestone complements earlier reimbursement pathways, such as those for CT-FFR, which has established CPT codes and reimbursement for evaluating CAD without invasive testing in the US. Besides direct reimbursement, there are routes AI can be cost-effective. A retrospective simulation study on lung cancer screening estimating the quality-adjusted life-years (QALYs) and lifetime costs of the diagnostic modalities showed that incremental cost-effectiveness ratio remained negative up to a threshold of USD 68 for the AI support.^108^ Another study evaluating a platform including 2 service lines and 5 individual packages, resulted in labour time reductions and delivery of a return of investment (ROI) of 451% over a 5-year period. The ROI was increased to 791% when radiologist time savings were considered. They reported that the most influential benefit of AI was the number of additional treatments performed because of AI identification of patients.^109^

### Con

While the potential benefits are compelling, there are significant challenges associated with AI adoption in cardiac imaging. High upfront costs, infrastructure upgrade requirements, and training for healthcare professionals are substantial. The process of creating a clinically viable AI product, including development, research, regulatory approval, design, and marketing/sales can cost many millions of dollars. Companies developing AI software are often supported by venture capital, resulting in relatively high cost of the product once clinical application is warranted. This must be considered in relationship to the reimbursement for cardiovascular imaging tests, such as CCTA. Although there are a few examples of reimbursable AI use, use-cases are still extremely limited, and little data is available on whether long-term benefits of AI will warrant continuous approval of reimbursement.^110^^,^^111^ In Europe, reimbursement remains less uniform, with each country adopting its own approaches. For example, CT-FFR solutions have seen reimbursement in several countries like the United Kingdom under NHS programmes, yet AI applications like plaque quantification often lack dedicated funding frameworks. van Kessel et al^112^ investigated 8 EU countries and their digital health reimbursement policies showing that countries have divergent approaches which also depends on how the health systems are financed. The fragmented European landscape contrasts with the more centralized coding system in the United States, emphasizing the need for harmonized policies to support broader adoption of medical AI. Different reimbursement strategies for AI can have an effect on the usage and willingness to adapt AI solutions. It is still unclear which reimbursement model is optimal for radiology-based AI applications.^110^^,^^111^

AI integration into clinical workflows can require ongoing technical support and maintenance, which adds to operational costs. Without large scale cost-effectiveness studies of AI applications in a clinical setting and data on long-term impact on patient outcomes, it can be difficult for institutes to justify the cost of implementing one or a set of AI applications. Additionally, the long-term cost-effectiveness of AI is uncertain, as continuous updates and monitoring are necessary to ensure optimal performance, which may offset some of the expected cost savings. The benefits of AI are not always immediately apparent, making it difficult to justify the significant investment required for widespread adoption. Cost-effectiveness of AI is highly dependent on the clinical setting and should be assessed locally. Challenges managing operational cost and implementation support are particularly pronounced outside of the usual training grounds of AI (mostly larger academic centres) in low-resource environments. These considerations increase the risk of widening the inequity gap already existing in healthcare with AI solutions being deployed and used mostly in high-income, metropolitan academic hospitals.^30^ Legal and ethical issues, such as liability for errors and data privacy, also contribute to the complexity of adoption and associated cost.

## Summary and conclusion

Some of the positives of AI are the often-excellent accuracy in image segmentation and quantification. Tools for automated measurements, enhanced image quality and worklist prioritization improve workflow efficiency, reducing reporting times and minimizing human error. Prognostic models show potential in predicting adverse cardiovascular outcomes, enabling personalized care. Regulatory agencies have begun approving AI tools while simultaneously reimbursement is becoming available, signalling growing trust in their safety and efficacy. However, there are some challenges AI has yet to overcome. Diagnostic and prognostic models often lack generalizability across diverse populations, risking biased outcomes and few studies show the effect on patient outcomes. AI tools face implementation hurdles in clinical practice due to a lack of information on cost-efficacy and IT integration. Regulatory oversight struggles to keep pace with AI’s rapid evolution, raising concerns about accountability and patient safety. Ethical dilemmas, including bias, data privacy and algorithmic transparency, remain unresolved. Interdisciplinary collaboration is needed to refine AI systems, assist with optimal implementation, ensure fairness, and optimize patient outcomes in cardiac imaging.
